# Affective pathways in human–AI interaction: the formation of willingness to communicate among Chinese KFL learners in AI-mediated informal digital learning of Korean

**DOI:** 10.3389/fpsyg.2026.1786682

**Published:** 2026-08-10

**Authors:** Yumei Jin, Byeonggon Min

**Affiliations:** Seoul National University, Seoul, Republic of Korea

**Keywords:** AI-mediated informal digital learning of Korean (AI-IDLK), Korean as a foreign language (KFL), L2 anxiety, L2 confidence, technology-enhanced language learning, willingness to communicate (WTC)

## Abstract

This study examines willingness to communicate (WTC) among Chinese learners of Korean in the context of AI-mediated informal digital learning of Korean (AI-IDLK) from an affective perspective. Moving beyond the assumption that L2 confidence and anxiety function as parallel affective predictors, the study suggests that these variables are related to WTC in different ways in contexts of human–AI interaction. Using an explanatory sequential mixed-methods design, this study collected survey data from 205 Chinese KFL learners and analyzed them using SPSS 27.0 and the PROCESS Macro, including descriptive statistics, exploratory factor analysis, reliability testing, Pearson correlation analysis, hierarchical multiple regression, and mediation analysis. Interview data from nine participants were examined through NVivo-supported reflexive thematic analysis. The quantitative results show that AI-IDLK is positively associated with WTC, with confidence showing the only significant indirect association, while anxiety does not exhibit a significant indirect effect. Qualitative findings further help explain these results by showing that confidence is associated with Psychological safety, Repetitive production practice, Personalized adaptive learning, Sustainable accessibility, and Discourse agency and engagement, whereas anxiety persists in relation to Uncertainty of AI responses, Uncontrollability of interaction, Uncritical praise, and the Gap with real interaction. Taken together, these findings suggest that the affective foundation of WTC in AI-IDLK may not be fully explained by the conventional view of confidence and anxiety as parallel affective predictors, and they call for a refinement of WTC theory that more explicitly accounts for the affective conditions of human–AI interaction.

## 1 Introduction

Affective factors have been consistently highlighted in second language (L2) research as core variables that explain learners’ language performance and communicative behavior (Dewaele and MacIntyre, 2014; MacIntyre et al., 1998; Zhang et al., 2018). Among these factors, confidence and anxiety function as primary determinants that directly drive learners’ cognitive engagement, risk-taking, and emotional regulation, exerting the most immediate influence on whether learners initiate or avoid communication in real interactional situations (Dewaele, 2019; Lin et al., 2025; Lee and Lee, 2021). These two affective variables have been treated as central mechanisms predicting the development of L2 willingness to communicate (WTC) (Cha and Tae-Young, 2013; Peng, 2012), and their functional roles within the broader WTC framework have largely been discussed under the assumption of relatively stable psychological and social conditions.

L2 WTC refers to learners’ momentary psychological readiness to engage in interaction using the target language (Cao and Philp, 2006), and the affective dimensions underlying this construct have been regarded as key indicators for explaining how communicative behavior is enacted (Freiermuth and Jarrell, 2006). According to the model proposed by MacIntyre et al. (1998), confidence facilitates WTC, whereas anxiety constrains it and traditionally these two variables have been understood within a relatively balanced structural relationship (Dewaele, 2019; Dewaele and MacIntyre, 2014; Peng and Woodrow, 2010). At the same time, as a construct defined in relation to readiness for communication at a particular moment of interaction, L2 WTC has increasingly been understood not simply as a fixed psychological disposition but as a dynamic construct that varies across situational conditions and momentary affective states (Lee and Chiu, 2024). This suggests that the functional relationship between confidence and anxiety should not be assumed to remain invariant across interactional contexts. However, this premise was constructed primarily in classroom-based or face-to-face interactional contexts where learners’ affective cues and social risks are assumed to remain relatively constant (Guan et al., 2024). Although contemporary learning ecologies have changed substantially, systematic examinations of whether these two key affective variables operate in similar ways in newly emerging interactional environments remain scarce.

In particular, with the recent emergence of generative artificial intelligence (AI) and large language models (LLMs), foreign language learning has shifted toward a new environment in which real-time interaction and personalized support are readily provided (Liu et al., 2025; Fathi et al., 2024; Kohnke et al., 2023). These changes are particularly pronounced in informal learning contexts in which learners move beyond the confines of formal instruction or classroom management systems and engage with generative AI tools (Liu et al., 2024a). In such contexts, AI-mediated informal digital language learning (AI-IDLL) offers affective and social conditions that are qualitatively distinct from those found in traditional computer-assisted language learning (CALL) or informal digital language learning (IDLL), thereby enabling forms of affective experience that were less accessible in earlier learning environments, such as heightened psychological safety, reduced burden of error, and expanded discourse agency (Belda-Medina and Calvo-Ferrer, 2022; Ebadi and Amini, 2024; Liang and Reiss, 2025).

Among these conditions, psychological safety is particularly significant because it may reshape not only the quality of AI-IDLL, but also the way participation itself is experienced. In the present study, psychological safety is treated not merely as a favorable feature of AI-IDLL, but as a condition that may reconfigure learners’ experience of participation itself. As originally conceptualized by Edmondson (1999), psychological safety refers to a condition in which individuals feel able to take interpersonal risks without fear of negative consequences. When applied to AI-IDLL, this concept helps explain how learners may engage in participation, feedback, and error management under conditions of reduced evaluative threat and interpersonal risk. If AI-IDLL reconfigures these interactional conditions, then the relative influence and functional relationship of confidence and anxiety may also differ from those assumed in traditional WTC models. From this perspective, confidence and anxiety may no longer operate in the manner presupposed in earlier frameworks; rather, their relative salience and structural relationship may be recalibrated within AI-IDLL environments. Nevertheless, empirical research that has structurally investigated how affective factors shape L2 WTC within AI-IDLL environments remains limited.

Against this backdrop, the present study takes MacIntyre et al.’s (1998) model of WTC as its theoretical point of departure and examines the structural pathways through which Chinese learners of Korean as a foreign language (KFL) develop L2 WTC via confidence and anxiety in the context of AI-mediated informal digital learning of Korean (AI-IDLK). Specifically, it investigates whether the relatively balanced functional relationship between confidence and anxiety assumed in traditional WTC models is maintained in AI-IDLK or takes on a different pattern in this context. This focus is significant because it examines AI-mediated language learning in relation to how human–AI interaction is associated with learners’ communicative readiness. Through this analysis, the study provides a more focused account of the affective mechanisms linking AI-IDLK to L2 WTC and broadens WTC and second language acquisition (SLA) research by offering evidence from the KFL context within the broader field of languages other than English (LOTE).

## 2 Literature review

### 2.1 AI-mediated informal digital learning of Korean (AI-IDLK)

Since the 1970s, CALL has been developed to emphasize learner autonomy and repetitive practice (Levy, 1997). This approach has expanded from classroom settings to digitally mediated informal learning, grounded in digital learning environments. Consequently, the concept of IDLL has emerged, referring to learners’ autonomous and informal language use (Lai et al., 2018). IDLL is defined as self-directed activities in which learners engage with the target language using online resources and digital tools (Lee, 2019; Zadorozhnyy and Lee, 2025). In Korean language learning, research has primarily focused on culturally mediated content, such as K-dramas and K-pop (Lee et al., 2025).

Traditional forms of IDLL have typically been input-driven or consumption-oriented, emphasizing learners’ passive reception and comprehension of information. The recent introduction of generative AI has addressed these structural limitations, enabling a new form of AI-IDLL in which learners co-construct linguistic knowledge through real-time interaction with AI (Ebadi and Amini, 2024). Liu et al. (2024a, 2024b) define this as a process in which learners autonomously engage in language learning outside the classroom using AI, exploring various learning affordances such as immediate feedback, error correction, and personalized suggestions.

AI tools support language learning by offering human-like linguistic responses and immediate feedback, enabling learners to produce and modify language in a quasi-authentic interactional environment that closely resembles real communication (Yang et al., 2025; Zhang et al., 2024). This interaction extends beyond simple input and output repetition, creating a cyclic self-regulatory process in which learners continuously monitor and revise their linguistic expressions, gradually internalizing language competence (Belda-Medina and Calvo-Ferrer, 2022). Additionally, AI-IDLL allows for repeated oral production and exploration of various discourse contexts in a low-risk environment, helping learners develop self-regulated performance routines for autonomous language use (Zhang and Liu, 2023). These features also suggest that informal digital language learning involves not only linguistic practice but also affective and motivational processes that shape learner engagement. In related IDLE research, Gao et al. (2025) showed that resilience and flow mediated the relationship between basic psychological needs and tertiary EFL learners’ engagement in informal digital learning of English.

Such interactive learning approaches have been discussed as extending beyond the acquisition of linguistic knowledge to support learners’ repeated use of the target language in authentic discourse contexts, thereby facilitating the gradual internalization of language use (Kohnke et al., 2023; Liu et al., 2024a). Against this broader IDLE background, recent AI-IDLE research has further examined how AI-mediated informal learning is related to learners’ beliefs, motivation, emotions, and engagement in English learning contexts. Wang et al. (2025) identified optimistic, critical, and hesitant belief types among Chinese EFL learners, showing that learners perceived AI-IDLE in differentiated ways, from strong expectations about its learning value to more cautious or uncertain views of its effectiveness. Wang et al. (2026) further showed that the ideal L2 self and positive achievement emotions, including enjoyment, hope, and pride, were positively associated with learners’ engagement in AI-IDLE, whereas the ought-to L2 self played a more peripheral role. Together, these studies suggest that recent research on IDLE and AI-IDLE has moved beyond questions of digital access and tool use toward the affective and motivational dimensions of learners’ informal language learning experiences.

However, existing research on AI-IDLL has focused largely on EFL learners. Although recent studies have broadened the discussion of IDLE and AI-IDLE to learners’ beliefs, motivation, emotions, and engagement, their empirical focus remains primarily situated within English learning contexts. This research tendency suggests that empirical findings and theoretical discussions have often been generalized from language-specific learning contexts. As a result, there remains limited comparative and contextualized understanding of how informal learning experiences and affective regulation processes shaped through human-AI interaction operate across learners of different foreign languages.

In this respect, the concept of AI-IDLL proposed by Liu et al. (2024a, 2024b) warrants further examination beyond English-centered research contexts and across a broader range of foreign language learning environments. Building on this line of inquiry, the present study conceptualizes AI-IDLK as self-directed and interactive learning activities in which learners of Korean engage with AI tools outside the boundaries of formal instruction or classroom management systems. Such activities include oral practice, error correction, question-answer interactions related to vocabulary and grammar, and writing, all of which emphasize language production through interaction.

### 2.2 L2 WTC

WTC was first introduced by McCroskey and Baer (1985) in the context of the first language (L1) and was subsequently extended to L2 research. MacIntyre and Charos (1996) empirically demonstrated WTC in L2 contexts, showing that it reflects psychological readiness to communicate rather than being determined solely by linguistic competence. MacIntyre et al. (1998) later defined L2 WTC as “a readiness to enter into discourse at a particular time with a specific person, using an L2” (p. 547), and proposed a six-layer pyramid model explaining how affective and cognitive factors influence the decision to speak. In this model, confidence and anxiety are key variables that directly affect communication: confidence facilitates speech, whereas anxiety leads to avoidance. More broadly, the pyramid model conceptualizes L2 WTC as the outcome of multiple hierarchically organized influences, ranging from relatively enduring social and individual factors to more immediate situational conditions.

Building on this framework, subsequent research has further shown that L2 WTC is not simply a stable personal trait but a situationally contingent construct shaped by learners’ immediate affective states, interlocutor relations, and contextual conditions (Lee and Chiu, 2024; Peng, 2012). This suggests that examining how proximal affective variables operate in evolving interactional environments is essential for understanding the formation of WTC. However, although a substantial body of research has examined L2 WTC in English language learning, corresponding research in Korean language education remains comparatively limited. This limitation is particularly evident in emerging interactional contexts where communication extends beyond formal instruction and is increasingly mediated by AI.

### 2.3 Confidence as a facilitator of L2 WTC

Research in SLA has consistently emphasized that learners’ language performance and communicative behavior are not merely products of linguistic competence, but are regulated by affective judgments and subjective self-perceptions (Dewaele, 2019). In particular, learners’ decisions to initiate an utterance or engage in interaction at a given moment are closely linked to how they perceive and evaluate their own language ability. Within this context, L2 confidence has been highlighted as a core affective judgment mechanism operating at a stage prior to actual language performance.

MacIntyre et al. (1998, 551) define L2 confidence as an individual’s belief in their ability to communicate effectively and adaptively in the target language, situating the construct within Bandura’s (1977) theory of self-efficacy. From this perspective, confidence is understood not as objective linguistic ability per se, but as a psychological variable reflecting how learners interpret their abilities and perceive them as usable communicative resources. In other words, L2 confidence functions both as a prerequisite for language performance and as an affective foundation that triggers communicative behaviors such as risk-taking, utterance initiation, and interactional persistence.

This theoretical position has been repeatedly supported by empirical research. Aziz and Rahadi Asih (2023) demonstrated that L2 confidence, as a self-efficacy based belief system, plays a structural role in shaping language performance and affective responses. Matsuda and Gobel (2004) reported that learners with lower confidence tend to avoid situations requiring the expression of complex meanings and exhibit inhibition during language production. This finding suggests that confidence functions not merely as an attitudinal variable, but as a substantive psychological filter that regulates communicative choices. In addition, Denies et al. (2015) and Edwards and Roger (2015) confirmed that learners with higher confidence participate more actively in learning activities and strengthen their WTC through repeated interactional experiences.

Importantly, the explanatory power of L2 confidence has been observed across diverse linguistic, cultural, and instructional settings, rather than being limited to a particular language or learning environment. Hashimoto (2002) reported that L2 confidence directly predicted both WTC and the frequency of language use among Japanese EFL learners. Using structural equation modeling, Khajavy et al. (2016) empirically demonstrated that L2 confidence serves as a significant direct predictor of WTC. In the context of Korean language education, Pyun et al. (2014) identified a significant positive relationship between learners’ confidence and WTC, while Lee et al. (2025) further showed that confidence constitutes a core affective predictor of WTC in Korean learning environments. Extending this line of inquiry, Lee and Lee (2020) demonstrated that L2 confidence continues to function as the strongest affective predictor of WTC even in digitally mediated communication contexts.

More recently, emerging research on AI-mediated language learning has suggested that such environments may provide conditions conducive to the development of learners’ confidence, particularly through repeated practice, immediate feedback, and responsive interactional support. For instance, Fathi et al. (2024) and Huang et al. (2025) reported that AI-supported learning environments can enhance learners’ confidence while promoting greater WTC. Similarly, Liu and Fan (2025) found that AI-generated speaking activities in classroom-based English learning fostered learners’ competence, confidence and WTC. Taken together, these findings suggest that even in AI-IDLL contexts, L2 confidence remains a central affective resource that supports learners’ WTC.

### 2.4 Anxiety as an inhibitory factor of L2 WTC

Foreign language anxiety (FLA), particularly speaking anxiety, has been widely discussed as a core affective factor in second language learning that not only undermines the quality of language performance but also constrains the psychological decision-making process through which learners determine whether to engage in communication. Horwitz et al. (1986) conceptualized speaking anxiety as a context-specific form of anxiety distinct from general test anxiety or social tension, and Horwitz (2001) further argued that such anxiety operates as a sustained state of tension arising from the interaction of cognitive appraisals and affective responses. Taken together, these perspectives suggest that anxiety is not a transient emotional reaction but a structural factor that systematically restricts learners’ communicative choice-making across the learning process.

From a WTC perspective, the importance of anxiety lies in its capacity to inhibit communicative engagement independently of language proficiency or learning motivation. MacIntyre and Gardner (1994) and Woodrow (2006) reported that speaking anxiety amplifies risk perception, thereby delaying utterance initiation and hindering the maintenance of interaction, while Teimouri et al. (2019) analyzed how elevated anxiety reinforces avoidance behaviors and excessive self-monitoring, resulting in a self-perpetuating cycle of reduced fluency. Taken together, these findings demonstrate that anxiety functions not as a consequence of performance, but as a psychological barrier that blocks WTC at a stage prior to actual language use.

This interpretation has been consistently supported by empirical research. Hashimoto (2002) and Khajavy et al. (2016) confirmed that anxiety exerts a significant negative effect on WTC even after controlling for language proficiency and learning motivation. This finding indicates that anxiety functions as an affective factor that directly constrains the transition from perceived communicative capability to communicative willingness. In other words, unlike confidence, anxiety can independently suppress WTC even in the absence of facilitative affective conditions.

At the same time, technology-mediated learning environments have been suggested to provide conditions under which such anxiety may be regulated. Satar and Özdener (2008) reported that text-based computer-mediated communication (CMC) significantly reduces anxiety, and similar affective effects have been observed in recent studies on AI-mediated language learning. Zhang et al. (2024) showed that an AI voice assistant alleviated anxiety and enhanced WTC among Chinese EFL learners, while Kim and Yuzhu (2024) confirmed that classroom-based AI chatbot activities reduced anxiety in KFL learning contexts. However, these discussions have largely been confined to teacher-led and structurally guided activities, leaving insufficient explanation of how anxiety operates in informal learning environments where learners autonomously regulate interactional intensity and exposure.

Taken together, prior research indicates that L2 confidence and anxiety function as two key proximal affective variables underlying learners’ WTC, with the former facilitating communicative engagement and the latter constraining it. Yet this body of research has been developed largely in relatively controlled instructional settings (Guan et al., 2024), leaving the operation of these affective mechanisms in AI-IDLL insufficiently examined. This gap is particularly important because such environments are becoming an increasingly salient part of learners’ everyday language use. In addition, although L2 WTC has been studied extensively in English language learning, corresponding research in languages other than English remains comparatively limited, with Korean language education representing a particularly underexplored case. From this perspective, examining how confidence and anxiety are related to WTC in AI-IDLK is important for clarifying the affective mechanisms underlying communicative readiness in this context and for extending WTC research beyond both conventional instructional settings and English-dominant learning environments.

### 2.5 Research questions and model

**Figure 1:**
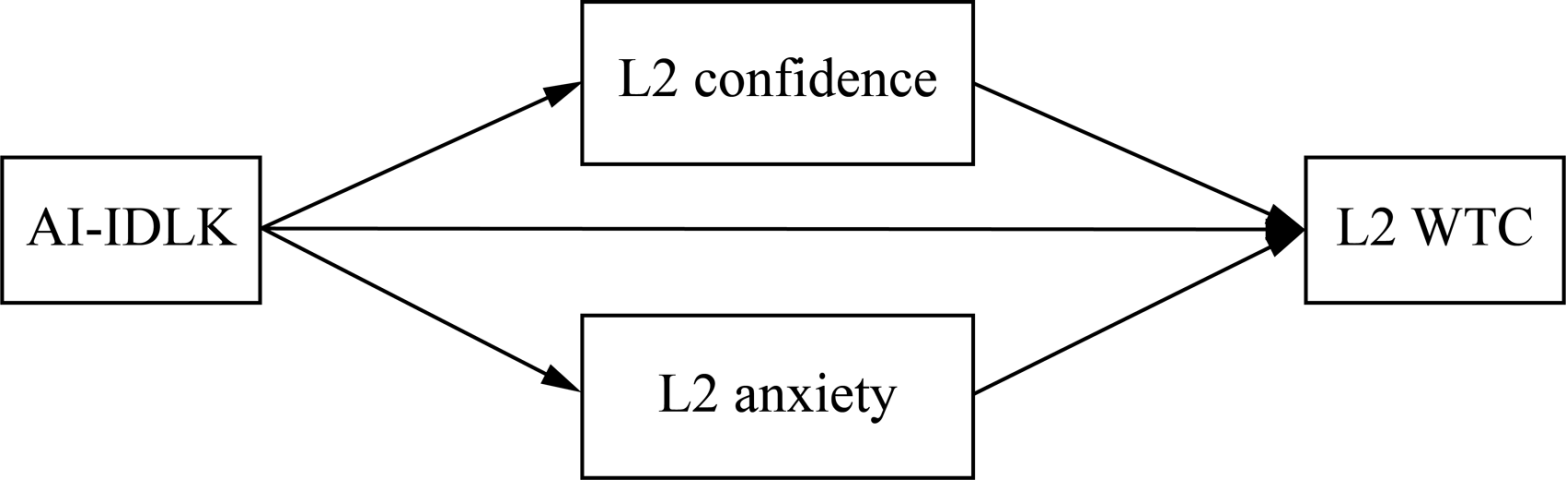
Research model.

Based on prior research, this study formulates the following research questions. Figure 1 illustrates the relationships among RQ1–RQ3, while RQ4 is examined separately through qualitative analysis.

*RQ1*. What is the relationship between AI-IDLK and Chinese KFL learners’ L2 WTC?

*RQ2*. What are the relative predictive powers of AI-IDLK, L2 confidence, and L2 anxiety on Chinese KFL learners’ L2 WTC?

*RQ3*. What mediating roles do L2 confidence and anxiety play between AI-IDLK and KFL learners’ L2 WTC?

*RQ4*. How do Chinese KFL learners describe the relationship between AI-IDLK and their L2 confidence, L2 anxiety and L2 WTC?

## 3 Methods

This study used an explanatory sequential mixed-methods design, combining quantitative and qualitative phases in sequence (Creswell and Clark, 2011). This approach integrates the generalizability of quantitative analysis with the contextual depth of qualitative inquiry, providing a more comprehensive understanding of the phenomenon under investigation.

### 3.1 Context and participants

The survey was conducted online from early June to late July 2025. Before the survey began, the study information sheet presented the eligibility criteria, and respondents were instructed to proceed only if they met these criteria. Two eligibility criteria were used for participant recruitment.

First, participants needed experience using AI tools such as ChatGPT and Gemini for autonomous Korean learning outside classroom-based formal curricula. In this context, “autonomous learning experience” referred to self-directed AI-mediated activities that supported the development of Korean communicative competence, including oral practice, error correction, vocabulary and grammar question-and-answer, writing, and AI-assisted resource searching. In particular, the inclusion of resource searching was based on the view that, in AI-IDLK, accessing explanations, examples, and learning materials through AI functions not as an end in itself, but as part of a broader meaning-focused learning process (Liu et al., 2024b). Accordingly, in the present study, resource searching was not treated as mere exploratory use, but was considered part of AI-mediated practices supporting Korean language learning and was therefore included within the scope of AI-IDLK.

Second, participants were required to have used AI-IDLK continuously for at least 3 months. This criterion was established based on prior evidence that new behaviors require approximately 66 days on average to become habitual (Lally et al., 2010), in order to distinguish sustained use from short-term exploratory or novelty-driven engagement. Together, these criteria ensured that respondents had relevant and sustained AI-IDLK experience, which was necessary for examining the relationships among AI-IDLK, L2 confidence, L2 anxiety, and L2 WTC.

Following this recruitment procedure, a total of 208 responses were collected. To ensure data quality, responses with the same option selected across all questionnaire items were treated as invalid and excluded. Accordingly, three responses were removed, resulting in a final sample of 205 valid cases for analysis. The final sample consisted of residents of mainland China studying KFL at 12 foreign language and comprehensive universities nationwide. Participants had a mean age of 22.7 years (SD = 3.1), and included 129 undergraduates (62.9%) and 76 postgraduates (37.1%). The mean duration of Korean language learning was 37.1 months (SD = 16.8). According to the Test of Proficiency in Korean (TOPIK), 20 learners (9.8%) were at the beginner level, 40 (19.5%) at the intermediate level, and 145 (70.7%) at the advanced level.

### 3.2 Quantitative data collection and analysis

The questionnaire first collected demographic information, including participants’ gender, age, university, education level, duration of Korean study, and Korean proficiency (TOPIK). Participants then completed 38 scale items assessing four key variables: AI-IDLK, L2 confidence, L2 anxiety, and L2 WTC (see Appendix A). The response format differed by construct. The AI-IDLK items were rated on a five-point frequency scale, ranging from 1 (never) to 5 (always), because they assessed the extent to which participants engaged in AI-IDLK practices. In contrast, the L2 confidence, L2 anxiety and L2 WTC items were rated on a five-point agreement scale, ranging from 1 (strongly disagree) to 5 (strongly agree), as these items measured participants’ perceived confidence, anxiety, and WTC.

The instruments used in this study were based on previously validated measures. Because the original scales were developed primarily in English-learning contexts, item wording was minimally adjusted to fit the present study, mainly by replacing references to English with Korean while preserving the original meaning of each construct. Specifically, AI-IDLK was measured using eight items adopted from Liu et al. (2024b). For example, one item was “I engage in Korean conversations with AI-powered chatbots on various topics to increase my exposure to Korean.” L2 confidence was measured with 12 items based on Aziz and Rahadi Asih (2023), with Item 6 reverse-coded as in the original scale. For example, “I know enough Korean to be able to comfortably.” L2 anxiety was measured with 10 items focusing primarily on speaking anxiety, based on the scale used by Yu et al. (2020). For example, “I worry about making mistakes when speaking in Korean.” L2 WTC was assessed with eight items drawn from Lee and Drajati (2019) to measure learners’ willingness to participate in communication in Korean. For example, “When you have a discussion with a small group of friends.”

This study used a multi-step quantitative analytical procedure, with all data analyzed in SPSS 27.0. Internal consistency was assessed using Cronbach’s *α*, and construct validity was examined through exploratory factor analysis (EFA) using principal component extraction. Prior to the EFA, the suitability of the data for factor analysis was assessed using the KMO measure and Bartlett’s test of sphericity. The cumulative variance explained was examined to assess how much of the total variance was accounted for by the extracted components. In addition, tolerance and variance inflation factor (VIF) values were calculated to assess potential multicollinearity among the variables and to ensure the stability of the regression analysis. Because all variables were measured through a single questionnaire at one time point, the possibility of common method variance was also examined as an initial diagnostic check using Harman’s single-factor test. In addition, to reduce this risk at the data collection stage, respondents were assured of anonymity and were provided with the same standardized instructions regarding the purpose of the survey and how to respond to the items.

For RQ1, Pearson correlation analysis identified correlations among key variables. For RQ2, hierarchical multiple regression analysis tested the predictive power of AI-IDLK, L2 confidence, and L2 anxiety on L2 WTC, controlling for demographic variables. For RQ3, the indirect effects of AI-IDLK on L2 WTC through L2 confidence and L2 anxiety were examined using the PROCESS Macro for SPSS (Model 4; Hayes, 2017). Mediation significance was determined by the 95% confidence interval (CI) from 5,000 bootstrapped resamples.

### 3.3 Qualitative data collection and analysis

Qualitative data were collected using purposive sampling to provide an in-depth interpretation of the quantitative analysis results. In particular, the qualitative phase was designed to provide a more in-depth examination of how learners experienced and interpreted AI-IDLK in relation to L2 confidence, L2 anxiety, and WTC, thereby helping to explain the patterns identified in the quantitative findings. Interview participants were drawn from the quantitative dataset, and nine respondents who had indicated their willingness to participate in follow-up interviews were purposively selected so as to reflect variation in affective responses and patterns of WTC associated with AI-IDLK. More specifically, the subsample was selected to include learners showing different combinations of confidence, anxiety, and WTC, rather than concentrating on a single affective or communicative profile. This participant selection was grounded in a sequential design in which the qualitative phase was used to further interpret the patterns identified in the quantitative findings. Appendix B contains detailed participant information. This study received approval from the appropriate Institutional Review Board (IRB No. 2510/003–021) and followed ethical procedures. Each interview was conducted in Mandarin via Zoom and lasted about 2 h. Before the interviews, participants submitted their AI learning dialogue records or screenshots, which were reviewed to examine their AI usage patterns and prompt practices. Based on this preliminary review and previous studies (Kim and Yuzhu, 2024; Liu et al., 2024a; Liu and Ma, 2024), a semi-structured interview protocol was developed (see Appendix C).

The audio recordings were first transcribed automatically using ClovaNote, after which the researcher manually corrected and supplemented the transcripts by checking them against the original audio. All materials were then translated into English, and the translations were reviewed through participant checking and review by two professional translators to ensure linguistic accuracy and consistency of meaning. RQ4 was analyzed following Braun and Clarke’s (2021) reflexive thematic analysis procedure, with NVivo used to support initial coding, the development of candidate themes, and the review, naming, and refinement of final themes. In line with this approach, themes were treated not as passively emerging from the data, but as interpretive constructions developed through the researcher’s active engagement with the interview materials. To make this interpretive process explicit, analytic decisions and reflexive considerations were documented throughout the analysis through memo writing and iterative theme review. This analytic process provided a contextual interpretation that complements the quantitative results (Creswell and Creswell, 2017). To enhance analytic transparency, Appendix D presents a condensed codebook together with illustrative excerpts from the interviews.

## 4 Results

### 4.1 Quantitative results

#### 4.1.1 Descriptive statistics, validity, and reliability of the scales

Table 1 presents the EFA results, descriptive statistics, and reliability estimates for each construct. Prior to examining the factor structure of the scales, the suitability of the data for factor analysis was assessed using the KMO measure and Bartlett’s test of sphericity. The results indicated a high level of sampling adequacy (KMO = 0.912), and Bartlett’s test of sphericity was statistically significant (χ^2^ = 5978.865, df = 703, *p* < 0.001), confirming that the inter-item correlation structure was appropriate for factor analysis. The EFA further supported the adequacy of the measurement structure. The cumulative variance explained by the four-factor solution was 60.616%, indicating an acceptable level of explanatory power for social science research. Because the explanatory contribution of subsequent factors was relatively limited, a four-factor structure comprising AI-IDLK, L2 confidence, L2 anxiety and L2 WTC was retained based on both theoretical coherence and the interpretability of the rotated factor-loading pattern.

**Table 1:** EFA, descriptive statistics, and reliability for each construct.

Construct	Item	FL	h2	Skew.	Kurt.	*M* (SD)	Construct *M* (SD)	α
AI-IDLK	1	0.751	0.660	−0.268	−1.016	3.39 (1.222)	3.38 (0.824)	0.806
	2	0.701	0.651	0.110	−1.091	2.88 (1.293)		
	3	0.683	0.563	−0.605	−0.673	3.51 (1.251)		
	4	0.514	0.517	−0.333	−0.864	3.20 (1.211)		
	5	0.709	0.538	−0.989	0.171	4.00 (1.109)		
	6	0.625	0.499	−0.341	−0.936	3.36 (1.285)		
	7	0.795	0.684	−0.570	−0.983	3.59 (1.360)		
	8	0.736	0.648	−0.183	−1.257	3.13 (1.378)		
L2 confidence	1	0.807	0.717	−0.651	0.154	3.85 (0.979)	3.54 (0.816)	0.936
	2	0.833	0.757	−0.579	−0.195	3.82 (1.001)		
	3	0.813	0.787	−0.212	−0.232	3.30 (1.041)		
	4	0.781	0.740	−0.320	−0.408	3.55 (1.045)		
	5	0.783	0.717	−0.164	−0.202	3.28 (0.989)		
	6*	−0.480	0.381	−0.281	−0.989	3.21 (1.237)		
	7	0.789	0.699	−0.414	−0.428	3.58 (1.057)		
	8	0.681	0.738	−0.288	−0.517	3.43 (1.090)		
	9	0.680	0.798	−0.277	−0.409	3.38 (1.072)		
	10	0.647	0.722	−0.716	0.069	3.78 (1.022)		
	11	0.635	0.785	−0.459	−0.497	3.59 (1.093)		
	12	0.565	0.769	−0.598	−0.277	3.69 (1.116)		
L2 anxiety	1	0.780	0.734	−0.126	−1.133	3.02 (1.325)	2.82 (0.998)	0.932
	2	0.806	0.779	0.074	−1.222	2.93 (1.350)		
	3	0.709	0.775	0.222	−0.996	2.68 (1.210)		
	4	0.769	0.745	−0.318	−0.986	3.27 (1.288)		
	5	0.580	0.619	−0.276	−0.801	3.23 (1.214)		
	6	0.739	0.792	0.280	−1.137	2.68 (1.322)		
	7	0.738	0.739	0.253	−0.996	2.65 (1.254)		
	8	0.748	0.733	0.224	−1.059	2.67 (1.262)		
	9	0.792	0.801	0.224	−0.945	2.56 (1.168)		
	10	0.720	0.700	0.385	−0.969	2.53 (1.270)		
L2 WTC	1	0.749	0.797	−0.446	−0.630	3.61 (1.090)	3.51 (0.898)	0.924
	2	0.754	0.814	−0.335	−0.678	3.48 (1.101)		
	3	0.619	0.720	−0.469	−0.554	3.68 (1.045)		
	4	0.734	0.792	0.120	−0.771	2.97 (1.129)		
	5	0.750	0.750	−0.538	−0.700	3.72 (1.137)		
	6	0.687	0.750	−0.392	−0.757	3.59 (1.145)		
	7	0.738	0.735	−0.588	−0.568	3.80 (1.083)		
	8	0.531	0.704	−0.102	−0.895	3.25 (1.152)		

As shown in Table 1, most factor loadings exceeded 0.50, and the communalities were generally acceptable, providing preliminary support for the adequacy of the measurement structure. In addition, the internal consistency of each construct was satisfactory, with Cronbach’s *α* values ranging from 0.806 to 0.936. Because all variables were measured through a single questionnaire at one time point, common method variance was also examined. Harman’s single-factor test showed that the first unrotated factor accounted for 34.878% of the total variance, suggesting that common method variance was not a serious concern in the present data. Taken together, these results suggest that the adapted scales used in this study showed acceptable psychometric properties and were appropriate for the subsequent analyses.

#### 4.1.2 Correlation analysis among AI-IDLK, L2 confidence, L2 anxiety, and L2 WTC

Table 2 presents the correlation analysis results. AI-IDLK had a significant positive correlation with L2 WTC (r = 0.375, *p* < 0.01), indicating that AI-IDLK was positively associated with learners’ L2 WTC. AI-IDLK also correlated positively with L2 confidence (*r* = 0.196, *p* < 0.01) but showed no significant relationship with L2 anxiety (*r* = 0.027, *p* > 0.05), suggesting that AI-IDLK was positively associated with confidence but not significantly associated with anxiety. L2 confidence correlated strongly and positively with L2 WTC (*r* = 0.646, *p* < 0.01), while L2 anxiety correlated negatively with L2 WTC (*r* = −0.468, *p* < 0.01). Additionally, L2 confidence and anxiety were significantly negatively correlated (*r* = −0.494, *p* < 0.01).

**Table 2:** Correlations among AI-IDLK, L2 confidence, L2 anxiety, and L2 WTC.

Variables	AI-IDLK	L2 confidence	L2 anxiety	L2 WTC
AI-IDLK	1	0.196**	0.027	0.375**
L2 confidence		1	−0.494**	0.646**
L2 anxiety			1	−0.468**
L2 WTC				1

**p < 0.01.

#### 4.1.3 Results of hierarchical regression analysis for predicting L2 WTC

A three-step hierarchical regression analysis was conducted to assess the relative explanatory power of demographic, linguistic, and affective variables on learners’ L2 WTC (see Table 3). In Model 1, demographic variables accounted for only 5% of the variance in L2 WTC and were not statistically significant (*R*^2^ = 0.056, *F* = 2.0, *p* > 0.05). Among these, only gender had a significant effect (*β* = −0.15, *p* < 0.05), with female participants (*M* = 3.44) reporting lower WTC than male participants (*M* = 3.73). In Model 2, language proficiency was entered as the sole predictor and was significant (*β* = 0.144, *p* = 0.040), but its explanatory power remained limited (*R*^2^ = 0.021). This result supports previous findings (MacIntyre et al., 1998; Peng, 2012; Yashima, 2002) indicating that, although language proficiency provides a foundation for WTC, it does not fully explain learners’ WTC.

**Table 3:** Hierarchical regression analysis predicting L2 WTC from AI-IDLK and affective variables.

Predictor variables	*B*	SE	*β*	*t*	*p*	Lower bound	Upper bound	Tolerance	VIF
Model 1									
Constant	3.309	0.380		8.707	0.000	2.560	4.059		
Gender	−0.307	0.147	−0.150	−2.083	0.039	−0.597	−0.016	0.915	1.093
Age	0.170	0.141	0.110	1.203	0.230	−0.109	0.448	0.564	1.772
Education level	−0.090	0.086	−0.098	−1.048	0.296	−0.260	0.079	0.542	1.843
Duration of Korean study	−0.031	0.140	−0.022	−0.222	0.824	−0.306	0.244	0.471	2.121
Korean proficiency (TOPIK)	0.240	0.130	0.177	1.848	0.066	−0.016	0.497	0.520	1.925
*R* = 0.238^a^, *R*^2^ = 0.057, Adjusted *R*^2^ = 0.033, *F* = 1.977, *p* = 0.071									
Model 2									
Constant	3.001	0.255		11.789	<0.001	2.499	3.503		
Korean proficiency (TOPIK)	0.196	0.095	0.144	2.072	0.040	0.009	0.382	1.000	1.000
*R* = 0.144^a^, *R*^2^ = 0.021, Adjusted *R*^2^ = 0.016, Δ*R*^2^ = –0.036, *F* = 4.293, *p* = 0.040									
Model 3									
Constant	1.239	0.335		3.701	<0.001	0.579	1.9		
AI-IDLK	0.316	0.055	0.290	5.784	<0.001	0.208	0.424	0.941	1.062
L2 confidence	0.516	0.063	0.468	8.132	<0.001	0.391	0.641	0.712	1.404
L2 anxiety	−0.220	0.051	−0.244	−4.324	<0.001	−0.32	−0.12	0.74	1.351
*R* = 0.725^a^, *R*^2^ = 0.525, Adjusted *R*^2^ = 0.518, Δ*R*^2^ = 0.504, *F* = 74.124, *p* < 0.001									

In Model 3, AI-IDLK, L2 confidence, and L2 anxiety were entered simultaneously as predictors. The model explained 52.5% of the variance in L2 WTC, indicating high explanatory power (*R*^2^ = 0.525, *p* < 0.001). All three variables were statistically significant. L2 confidence (*β* = 0.468, *p* < 0.001) was the strongest positive predictor, followed by AI-IDLK (*β* = 0.290, *p* < 0.001), which showed a significant positive coefficient in relation to WTC. In contrast, L2 anxiety (*β* = −0.244, *p* < 0.001) showed a significant negative coefficient, indicating that lower anxiety corresponded to higher reported WTC. In addition, all tolerance values exceeded 0.20 and all VIF values were below 5.00, indicating the absence of multicollinearity across the regression models. Residual plots suggested no serious departures from linearity or homoscedasticity and the residuals appeared to be approximately normally distributed. Furthermore, the 95% confidence intervals for the significant predictors did not include zero, providing additional support for the statistical significance of the regression coefficients.

#### 4.1.4 Mediation effects of L2 confidence and anxiety in the relationship between AI-IDLK and L2 WTC

Table 4 shows that AI-IDLK had a significant association with L2 WTC (effect = 0.316, *p* < 0.001), and the total effect was also significant (effect = 0.409, *p* < 0.001). Of the two affective variables, only L2 confidence demonstrated a significant indirect effect (effect = 0.100, 95% CI [0.024, 0.192]), while L2 anxiety did not (effect = −0.007, 95% CI [−0.052, 0.036]). These results indicate that only L2 confidence showed a significant indirect effect in the relationship between AI-IDLK and L2 WTC, whereas no significant effect was observed for anxiety.

**Table 4:** Mediation analysis of the effects of AI-IDLK on L2 WTC via affective variables.

Pathway	Effect	SE	95% Confidence Interval (CI)	Significance
AI-IDLK → L2 WTC	0.3159	0.0546	(0.2082, 0.4236)	0.0000
AI-IDLK → L2 confidence → L2 WTC	0.1001	0.0426	(0.0236, 0.1915)	
AI-IDLK → L2 anxiety → L2 WTC	−0.0072	0.0215	(−0.0516, 0.0356)	
Total Effect	0.4088	0.0709	(0.2689, 0.5486)	0.0000

**Figure 2:**
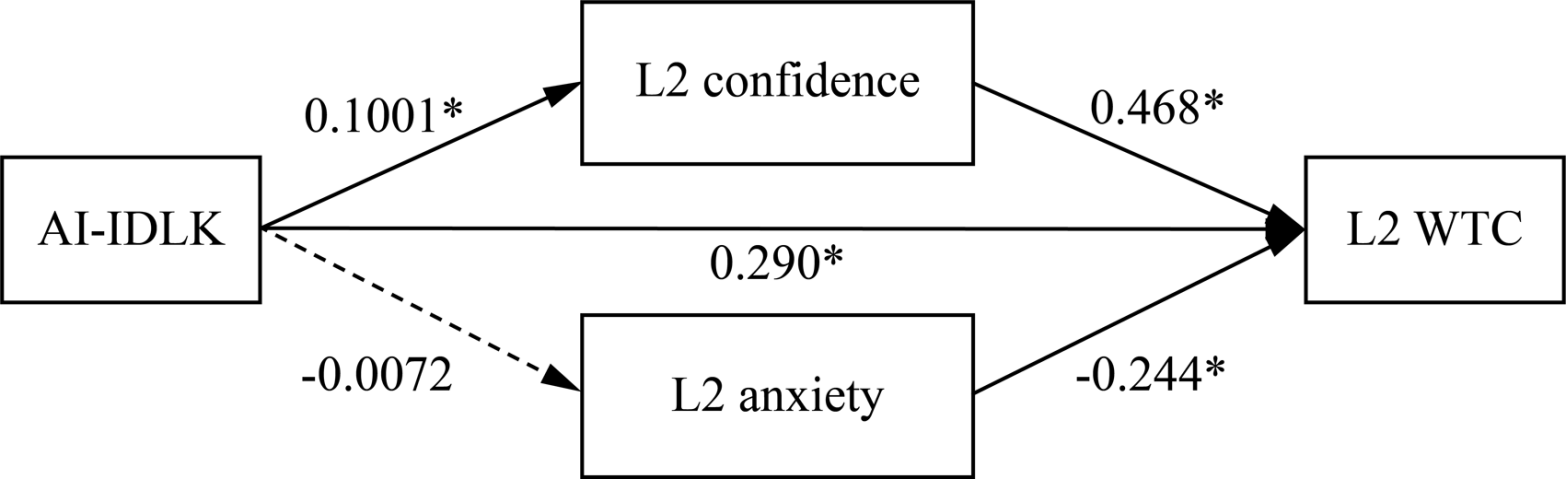
Final model.

Synthesizing these results, a final model was developed that integrates the main relationships among AI-IDLK, L2 confidence, L2 anxiety, and L2 WTC (see Figure 2)^1^.

The quantitative analysis results are presented in accordance with the research questions. For RQ1, AI-IDLK was significantly associated with the L2 WTC of Chinese KFL learners (*β* = 0.290, *p* < 0.001), indicating that AI-IDLK is closely associated with increased WTC levels. For RQ2, L2 confidence was the strongest predictor (*β* = 0.468, *p* < 0.001), while AI-IDLK (*β* = 0.290, *p* < 0.001) and L2 anxiety (*β* = −0.244, *p* < 0.001) also showed significant predictive power (*R*^2^ = 0.525). These findings indicate that affective factors and AI-IDLK together contribute to L2 WTC. For RQ3, AI-IDLK had a significant indirect effect on L2 WTC through L2 confidence (*β* = 0.1001, *p* < 0.05), but the indirect effect through L2 anxiety was not significant (*β* = −0.0072, *p* > 0.05). These results show that L2 confidence is a key indirect pathway, whereas anxiety does not serve as a significant mediator.

### 4.2 Qualitative results

The qualitative analysis addressed RQ4 and offered a detailed interpretation of the quantitative findings. The analysis was structured around three thematic dimensions: the nature of AI-IDLK learning experiences, the L2 confidence enhancement pathway, and the L2 anxiety persistence pathway.

#### 4.2.1 Nature of AI-IDLK learning experiences

Participants reported that their motivation to engage in AI-IDLK primarily arose from the need to address limited learning resources and opportunities. Korean language education in China generally lacked qualified teachers, language institutes, and online courses, with availability varying by region and institution. As a result, learners described deficiencies in their learning environments. For example, P3, P4, and P7 reported uncertainty about how to study independently, as expressed in the comment “I did not really know how to study on my own” (P3). P1, P2, and P5 noted insufficient opportunities for practice, reflected in “I felt that the amount of practice in class was insufficient” (P1). Likewise, P6 and P8 pointed to restricted speaking opportunities due to teacher-centered instruction, illustrated by “Classes were teacher-centered, so I hardly had a chance to speak myself” (P6). Thus, learners viewed AI-IDLK not only as a supplementary tool but also as an alternative learning space accessible anytime and anywhere.

**Figure 3:**
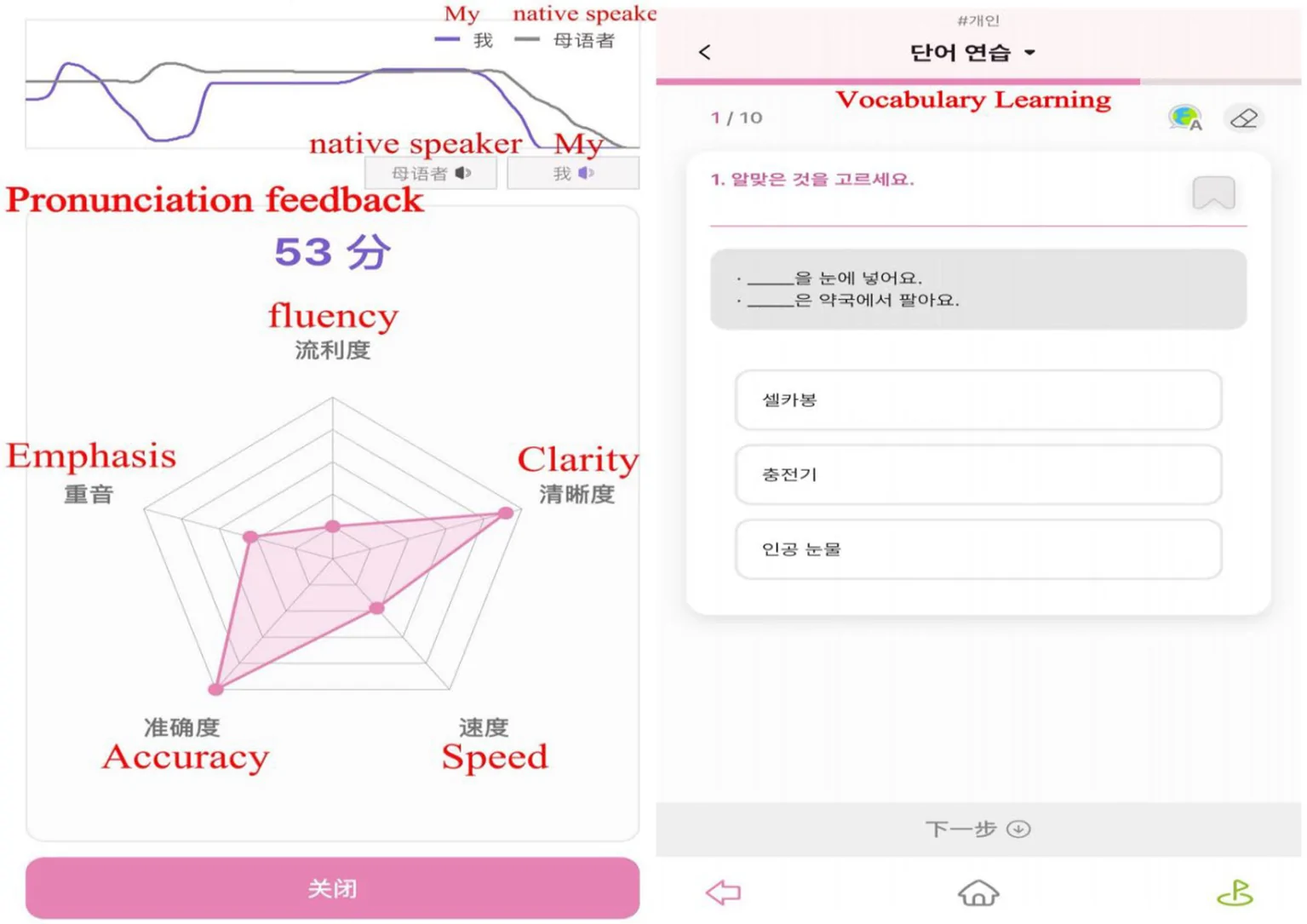
AI-assisted pronunciation and vocabulary learning (“KOKOA”; KOKOA - TOPIK & Korean, n.d.).

**Figure 4:**
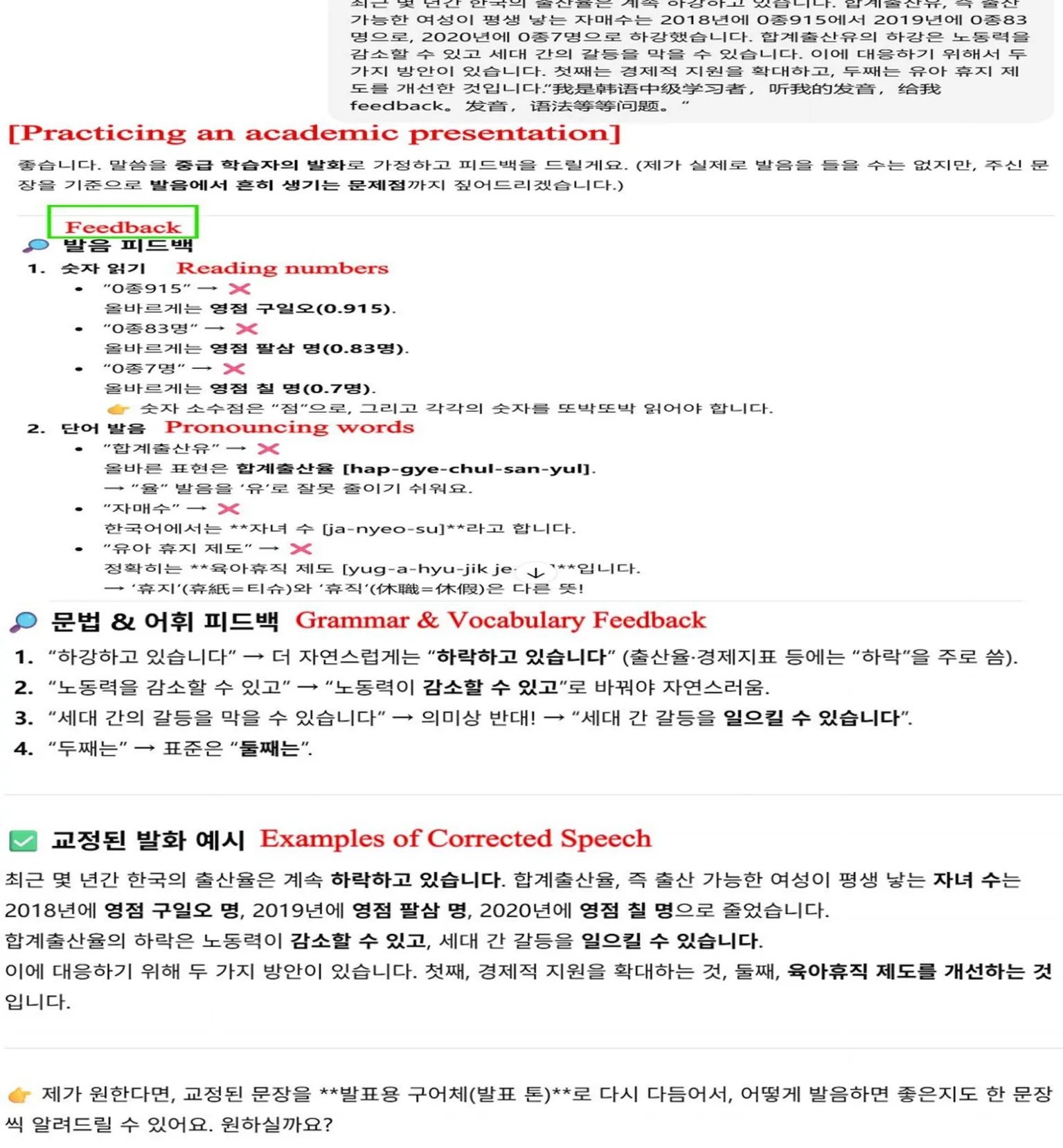
AI-assisted academic presentation practice (“ChatGPT”; ChatGPT, n.d.).

In the AI-IDLK learning experiences, beginner and intermediate learners primarily used the tools for activities that integrated receptive and productive language skills, such as pronunciation recognition, error correction, grammar learning, vocabulary expansion, and scenario-based practice (see Figure 3), as well as scenario-based practice. In contrast, advanced learners engaged in complex simulation tasks, including discussion, academic presentation (see Figure 4), academic writing, and data interpretation, and demonstrated higher-level language functions such as meaning negotiation, logical development, discourse organization, and revision.

In the Chinese context, classes or training sessions focused on prompt-writing methods or AI utilization strategies are relatively scarce. Consequently, several learners indicated a need for systematic instruction. Many expressed a desire for structured guidance on effective AI use (P1, P2, P4, P5, P6, P7, P8), exemplified by the comment, “I wish there were classes that teach how to use AI more effectively” (P6). Others emphasized the importance of learning prompt strategies (P2, P3, P4, P5, P9), as illustrated by the remark, “I want to learn how to write more diverse prompts” (P9). These statements show that learners are not passive consumers of technology; instead, they seek to expand their AI learning with more professional and organized support. AI-IDLK served as an alternative learning mechanism that addressed limited institutional resources and as a platform where learners actively sought and requested new learning opportunities. This indicates that AI-IDLK, beyond functioning as a technological aid, is a learning context with the potential to increase Chinese KFL learners’ actual language use opportunities and learner autonomy.

#### 4.2.2 The L2 confidence enhancement pathway

The quantitative analysis confirmed that AI-IDLK was positively related to L2 WTC, with L2 confidence showing a significant indirect effect in this relationship (see Figure 2). The qualitative data also consistently supported these findings. Learners’ increased confidence was not simply the result of more utterances; instead, it developed through the combination of various AI-provided learning conditions, which was linked to actual WTC.

Learners identified psychological safety as essential for increasing confidence. For example, statements such as “AI does not laugh even if I make mistakes, so I do not have to worry about being wrong” (P3) and “I used to be afraid to speak because of the pressure to do well, but I became more confident while practicing with AI” (P2) indicate that an environment without evaluation anxiety encouraged more frequent speaking attempts and improved confidence.

Building on this sense of safety, learners increased their confidence through repetitive production practice. Statements such as “As I practiced repeatedly, I gained confidence that I could speak in Korean” (P4), “In class, there are few opportunities to speak, but with AI, I can keep talking as much as I want, so my expressive ability has improved” (P7), and “While preparing for an interview, I practiced with AI, and by repeatedly answering questions, I became more fluent and confident… I felt that I could speak without nervousness even in the actual interview situation” (P6) indicate that repetitive production practice was linked to a foundation for actual communicative participation.

Learners identified personalized adaptive learning as a qualitative factor that enhanced their confidence. In classroom instruction, individualized learning and feedback are limited; however, AI consistently provided support tailored to each learner’s level and context. For example, participants stated, “Through simulated debates with AI, I learned various expression strategies and wanted to apply them in real discussions” (P1), “In textbooks there is only one correct answer, but AI gives accurate responses suited to my utterances… It is my one-on-one teacher” (P4), and “When I practiced conversations assuming translation situations, it taught me professional terms and context-specific expressions… I felt that I could explain things in Korean in an actual translation setting” (P9). These statements illustrate how learning experiences were connected to WTC.

Personalized adaptive support, combined with sustainable accessibility, transformed confidence into a long-term asset. Statements such as “I can practice Korean regardless of day or night” (P3) and “I can talk in Korean whenever I want… as it became a habit, Korean came out unconsciously” (P7) indicate that removing temporal and spatial constraints enabled sustained speaking. Additionally, the statement “As I practiced academic topics with AI, I became able to speak naturally even in academic presentation situations” (P8) shows that WTC extended beyond everyday conversation to academic presentation contexts.

Discourse agency and emotional engagement served as key pathways for increasing L2 confidence. Through interactions with AI, learners viewed themselves as active participants in language use and reconstructed their identities through speech. For example, “Since AI followed the flow of my utterances, I felt that I was controlling the direction of the conversation” (P6) and “When I explained legal cases in Korean by myself, I gained confidence that I could think and express myself in Korean” (P9) indicate that learners internalized confidence by gaining control over language use. These agentive speaking experiences promoted immersion in conversations, as shown by responses such as “When I practiced with AI whose voice was similar to my favorite idol’s, I felt immersed as if I were having a real conversation” (P5) and “I enjoyed talking with AI so much that I kept speaking in Korean without being aware of time” (P7). These examples suggest that emotional immersion supported sustained speaking and was linked to higher WTC.

#### 4.2.3 The L2 anxiety persistence pathway

AI-IDLK offered learners a new environment for practicing speech; however, anxiety remained evident. Learners identified the unpredictability of AI responses as a primary reason for this outcome. For example, participants reported, “When AI got something wrong, I thought it was my mistake, so I could not continue speaking” (P2); “AI is not always accurate, so I feel anxious about whether I might be learning something incorrectly” (P8); and “AI said it was correct, but it turned out to be wrong. When my teacher later said something different, I felt that everything I had practiced was wrong and stopped speaking” (P4). These statements indicate that anxiety was closely tied to learners’ willingness to participate in speaking activities.

Learners identified the uncontrollability of interaction as a key factor associated with the persistence of their anxiety. For example, participants stated, “When AI suddenly responds in a different direction while I’m studying, I lose the desire to continue speaking” (P5), and “AI sometimes does not answer my questions, which makes me feel frustrated” (P9). These statements indicate that unpredictability was linked to a lower willingness to continue speaking.

Learners also reported that uncritical praise from AI was linked to greater anxiety. While positive feedback offered temporary reassurance, the lack of specific comments on detailed errors led them to view it as “unverified praise.” For example, participants stated, “Even when my grammar might be wrong while speaking, AI just says I did well, so I’m not sure if I actually did it correctly” (P6), and “AI keeps saying I did well no matter what I do, so I could not tell whether it really meant I passed or it just overlooked my mistakes” (P3). These statements indicate that excessive kindness was experienced as inadequate evaluation, which was linked to stronger anxiety.

**Figure 5:**
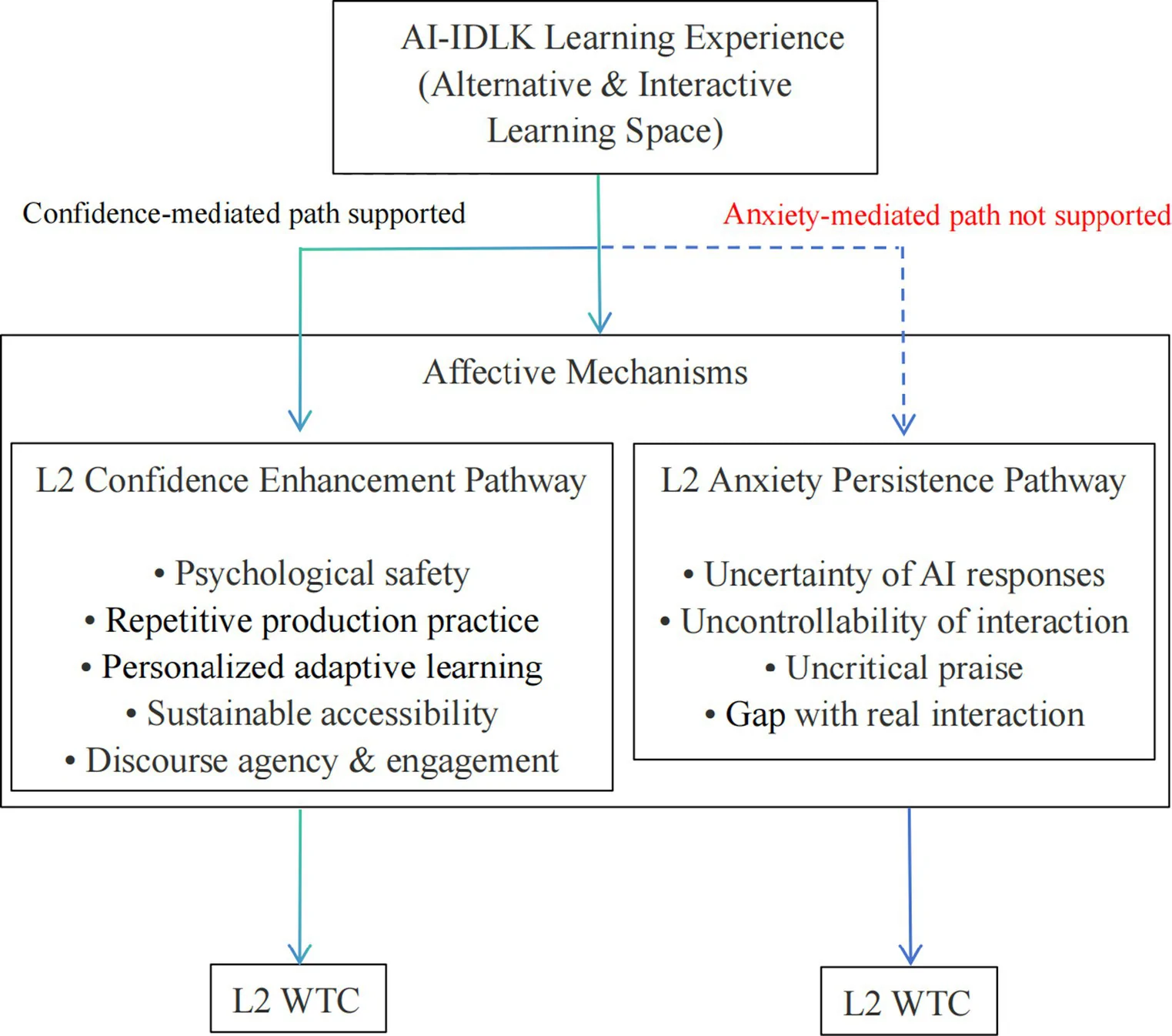
Categorized affective pathways of L2 confidence and anxiety in AI-IDLK.

Finally, the gap between AI interaction and real-life communication was associated with persistent cognitive anxiety among learners. For example, participants stated, “AI only answers what I ask, but when I talk with people, unexpected questions make me nervous” (P7), and “I feel fine when practicing with AI, but I get a bit anxious when actually speaking” (P4). These comments show that anxiety did not originate from new external factors; rather, it persisted because the sense of control experienced in AI contexts coexisted with the social complexity of real-life situations. AI-IDLK was associated with learners’ psychological stability while also coexisting with a persistent form of cognitive-situational anxiety, characterized by cognitive uncertainty and social transfer tension. The findings show that confidence and anxiety coexist as distinct emotional mechanisms that contribute to WTC in different ways rather than in opposition. This suggests that AI-IDLK may be associated not only with differences in emotional intensity but also with shifts in the affective conditions underlying WTC, with important theoretical implications. Figure 5 illustrates this distinction, presenting confidence as a facilitative pathway and anxiety as an inhibitive pathway.

## 5 Discussion

The quantitative results for RQ1 indicate that AI-IDLK was positively related to Chinese KFL learners’ L2 WTC. This finding suggests that AI functions not merely as a supplementary learning tool but as an interactional environment that may support learners’ communicative participation. In MacIntyre et al.’s (1998) WTC model, actual communication is triggered by learners’ immediate interactional conditions. From this perspective, AI-IDLK, which provides repeated and contextualized opportunities for oral practice can be interpreted as an environment that may alter the conditions under which WTC is activated. These results are consistent with prior research demonstrating that repetitive practice and contextualized interaction are positively related to WTC (Belda-Medina and Calvo-Ferrer, 2022; Liu and Fan, 2025). They further indicate that such effects are observable in KFL contexts that have received comparatively less scholarly attention, thereby extending the applicability of WTC theory beyond LOTE learning contexts.

The regression analysis for RQ2 further clarifies the affective structure underlying WTC in the AI-IDLK context. Among the three predictors, L2 confidence emerged as the strongest determinant of WTC, consistent with prior SLA research that positions confidence as a core proximal variable in WTC formation (Hashimoto, 2002; Khajavy et al., 2016; Mulyono and Saskia, 2021). At the same time, AI-IDLK itself showed a significant positive coefficient in relation to WTC (Fathi et al., 2024; Kim and Yuzhu, 2024), indicating that the learning environment may be linked to WTC independently of learners’ affective states. In contrast, L2 anxiety functioned as an inhibitory factor, aligning with established findings in the WTC literature (Cha and Tae-Young, 2013). However, subsequent mediation analyses clearly suggest that these two affective variables do not operate on WTC in the same manner. It is also noteworthy that the effects of gender and Korean proficiency became attenuated once the affective variables were entered into the model. This pattern is in line with prior WTC research, which has emphasized the more proximal role of affective variables, particularly communication confidence and anxiety, in relation to learners’ WTC (Hashimoto, 2002; Khajavy et al., 2016; Peng and Woodrow, 2010).

The mediation and qualitative analyses for RQ3 and RQ4 indicate that the substantive affective pathway linking AI-IDLK to WTC was evident only through confidence. In the present study, AI-IDLK in the quantitative model was treated primarily as learners’ behavioral engagement in AI-mediated informal learning activities, whereas the qualitative strand further illuminated the affective and interactional qualities through which such engagement was experienced. In the qualitative findings, AI-IDLK was associated with learners’ confidence through psychological safety, repetitive production practice, personalized adaptive learning, sustainable accessibility, and discourse agency and engagement. Under these conditions, learners came to perceive language use not as a one-time evaluative event or a high-risk performance, but as a communicative resource that could be gradually accumulated. This process was consistent with an indirect pathway through which confidence was linked to actual communicative behavior (Fathi et al., 2024; Zhang et al., 2024). These findings empirically support the theoretical conceptualization of the WTC model proposed by MacIntyre et al. (1998), which posits a close linkage between proximal affective variables and communicative behavior, while also demonstrating that the strength and mode of operation of these affective mechanisms may vary depending on the learning environment.

In contrast, the anxiety pathway was not significant, constituting the most important theoretical contribution of the present study. Whereas previous classroom-based AI research reported concurrent increases in confidence and decreases in anxiety (Kim and Yuzhu, 2024; Zhang et al., 2024), no significant association with lower anxiety was observed in the AI-IDLK context. Qualitative evidence suggests that, in the absence of teachers functioning as social buffers, anxiety persisted in relation to uncertainty of AI responses, uncontrollability of interactions, uncritical praise, and the gap with real interaction. As a result, despite the higher salience of confidence, anxiety continued to independently relate to lower WTC, confirming that the two affective variables operated through distinct pathways rather than in parallel.

The findings of this study point to the need for a more contextually grounded interpretation of the affective layer of the WTC model proposed by MacIntyre et al. (1998). In this model, confidence and anxiety are positioned at the same affective level proximal to communicative behavior and are conceptualized as influencing the immediate likelihood of WTC. However, the original pyramid model does not clearly specify whether these proximal affective variables operate with comparable weight across learning contexts, or whether their relative salience is conditioned by specific contextual features. In the present study, confidence was more closely aligned with both AI-IDLK and WTC, whereas anxiety remained negatively associated with WTC but was not comparably related to AI-IDLK in this sample. This pattern does not diminish the theoretical significance of anxiety within the WTC framework. Rather, it suggests that, in AI-mediated informal learning, the experiential conditions associated with AI-IDLK may be more readily linked to the development of confidence than to the attenuation of anxiety. Accordingly, the affective layer of WTC may be more appropriately understood as context-sensitive in its configuration, with the relative prominence of proximal affective conditions varying according to the interactional context.

From a theoretical standpoint, this study extends current understanding of the affective dimension of WTC demonstrating how such contextual variation becomes visible in AI-mediated informal language learning. More specifically, the present findings indicate that, in the context of AI-IDLK, WTC was more closely related to confidence formation than to anxiety reduction. This indicates that AI-mediated informal learning may not shape all affective conditions underlying WTC to the same extent, but may be more closely related to those that support the development of learners’ WTC. In this sense, the study broadens the explanatory scope of WTC theory, which has been developed largely in classroom-based human-to-human interaction, by demonstrating the need to account for how the affective conditions underlying WTC are configured in informal human-AI interaction. More broadly, the findings offer important insights into how the affective basis of WTC may be reconfigured in context-specific ways within the ongoing digital transformation of language learning.

## 6 Conclusion

### 6.1 Summary of research findings

This study adopted a mixed-methods approach to investigate how confidence and anxiety, two core affective variables, function in relation to the WTC of Chinese KFL learners within the context of AI-IDLK. Addressing four research questions, the study examined the relationship between AI-IDLK and L2 WTC, the relative predictive strength of AI-IDLK, confidence, and anxiety, the indirect associations of confidence and anxiety in the relationship between AI-IDLK and WTC, and the ways in which AI-IDLK was related to these affective variables. The quantitative findings showed that AI-IDLK was positively related to WTC, with confidence emerging as the strongest predictor among the three variables. Mediation analyses further indicated that confidence showed the only significant indirect association between AI-IDLK and WTC, whereas anxiety did not show a significant indirect association in this sample.

Qualitative findings provided process-level explanations for these results, elucidating how AI-IDLK was associated with confidence while allowing anxiety to persist. Specifically, confidence was associated with psychological safety, repetitive production practice, personalized adaptive learning, sustainable accessibility, and discourse agency and engagement. In contrast, anxiety persisted in relation to uncertainty of AI responses, uncontrollability of interactions, uncritical praise, and the gap with real interaction. Together, these findings indicate that AI-IDLK does not uniformly relate to learners’ affective states but appears more closely linked to facilitative affect while leaving inhibitory affect largely unchanged.

This study makes two interrelated contributions. Theoretically, it suggests that facilitative affect (confidence) and inhibitive affect (anxiety) may not be equally implicated in WTC across all learning conditions. In the present study, AI-IDLK was more closely associated with confidence formation than with anxiety reduction, indicating that these two affective variables were not related to AI-IDLK in the same way in relation to WTC. This does not diminish the importance of anxiety within the WTC framework; rather, it suggests that, in AI-mediated informal learning, the conditions associated with AI-IDLK may be more conducive to strengthening confidence than to reducing the anxieties that remain relevant to WTC. In this sense, the findings call for a more nuanced understanding of how confidence and anxiety have been jointly positioned as proximal affective variables within the WTC model proposed by MacIntyre et al. (1998). Accordingly, the affective layer of the pyramid model may be better understood as context-sensitive, with the relative importance of proximal affective conditions varying across interactional and learning contexts. This extends the scope of WTC theory by offering a more contextually grounded account of how affective conditions underlying WTC may be configured in AI-mediated interaction contexts.

Contextually, the study extends predominantly EFL-centered research on WTC and affective variables to the underexplored domain of KFL and more broadly to LOTE. Rather than positioning KFL as a special case, the findings suggest that AI-IDLK can function as a core learning ecosystem that is linked to learner autonomy and communicative engagement even in resource-constrained environments. Importantly, the identification of variation in how affective conditions were related to WTC in AI-IDLK provides new insight into how WTC may be differently organized when communication occurs through human–AI interaction rather than traditional human-to-human interaction.

### 6.2 Research limitations

Despite these contributions, several limitations should be noted. First, because this study was based on cross-sectional survey data, the findings are best understood as identifying structural relationships among the variables at the time of data collection, rather than capturing how these relationships may develop over time. Moreover, because all variables were measured through self-report in a single questionnaire, the possibility of common method variance cannot be entirely ruled out. Although Harman’s single-factor test was used as an initial diagnostic check and procedural steps were taken to reduce this risk during data collection, these measures should not be regarded as providing a strong control for such bias. Second, the sample included a relatively high proportion of advanced-level TOPIK learners, which may have limited the extent to which proficiency-related variation could be examined in greater detail. Because affective profiles at higher proficiency levels may differ from those of less proficient learners, caution is needed in extending the present findings to AI-IDLK more generally. Third, as the study focused on Chinese learners of Korean engaged in AI-IDLK, caution is needed in generalizing the findings to other learner populations, target languages, or instructional contexts.

### 6.3 Future research directions

Future research could extend the present findings in several ways. Longitudinal designs would be useful for examining how the relationships among AI-IDLK, confidence, anxiety, and WTC develop over time, rather than only identifying their structural associations at a single point. In addition, future studies with more proficiency-balanced samples could clarify whether the affective pathways identified in this study operate differently across beginner, intermediate, and advanced learners. Further research involving more diverse learner populations, target languages, and instructional contexts would also help determine whether the affective configuration observed in AI-IDLK is specific to Chinese KFL learners or reflects broader patterns in AI-mediated informal language learning. Together, such research would help extend the applicability of the present findings and further clarify the affective dynamics underlying L2 WTC in AI-mediated language learning contexts.

## Data Availability

The de-identified data supporting the conclusions of this article will be made available by the authors upon reasonable request, subject to ethical considerations.

## Footnotes

1 The coefficients for the paths leading to L2 WTC (0.290, 0.468, and −0.244) are standardized regression coefficients (*β*) from the hierarchical regression analysis reported in Table 3, whereas the values for the two indirect paths (0.1001 and −0.0072) are unstandardized indirect effects (B) from the PROCESS analysis reported in Table 4.
