# Supplementary material for: Affective pathways in human–AI interaction: the formation of willingness to communicate among Chinese KFL learners in AI-mediated informal digital learning of Korean
Source: Front Psychol. 2026 Aug 10;17:1786682. doi: 10.3389/fpsyg.2026.1786682 (PMC13500278; doi:10.3389/fpsyg.2026.1786682)
Supplement: Supplementary file 1 [file Supplementary_file_1.docx]

**Appendices**

**Appendix A: The 38-item questionnaire**

| ***AI-mediated informal digital learning of Korean (AI-IDLK)*** | |
| --- | --- |
| 1 | I engage in Korean conversations with AI-powered chatbots on various topics to increase my exposure to Korean. |
| 2 | I use AI-powered speech recognition tools to practice speaking Korean and receive immediate feedback on my fluency and intonation. |
| 3 | I play AI-powered language learning games to expand my Korean vocabulary. |
| 4 | I interact with AI-powered chatbots to acquire personalized Korean learning tips and strategies. |
| 5 | I use new AI technologies to find and obtain Korean learning resources. |
| 6 | I engage in discussions with AI-powered chatbots to deepen my understanding of Korean language and culture. |
| 7 | I interact with AI-powered chatbots to practice my Korean writing skills beyond the classroom. |
| 8 | I use AI technologies to simulate real-life Korean language use situations. |
| ***L2 Self-Confidence*** | |
| 1 | I believe that I am capable of reading and understanding most texts in Korean. |
| 2 | I feel that I can understand someone speaking Korean quite well. |
| 3 | I know enough Korean to be able to communicate comfortably in Korean. |
| 4 | I believe that I know enough Korean to speak correctly. |
| 5 | I am confident in my ability to speak Korean correctly. |
| 6* | Sometimes I feel uncomfortable speaking in Korean because of my accent. |
| 7 | I believe that my knowledge of Korean allows me to cope with most situations where I have to use Korean. |
| 8 | When I need to make a telephone call in Korean, most of the time, I am confident that I can do it. |
| 9 | Every time that I meet a Korean-speaking person and I speak with him/her in Korean, I feel easy and confident. |
| 10 | In a restaurant, I feel confident when I have to order a meal in Korean. |
| 11 | I feel confident and relaxed when I have to ask for directions in Korean. |
| 12 | I feel comfortable when I speak Korean among friends where there are people who speak Korean. |
| ***L2 Anxiety*** | |
| 1 | I worry about making mistakes when speaking in Korean. |
| 2 | I start to panic when I have to speak without preparation in Korean. |
| 3 | When speaking Korean, I can get so nervous I forget things I know. |
| 4 | It embarrasses me to initiate a communication in Korean. |
| 5 | Even if I am well prepared for Korean speaking, I feel anxious about it. |
| 6 | I can feel my heart pounding when I'm going to be asked to respond in Korean. |
| 7 | I always feel that others speak Korean better than I do. |
| 8 | Conversation in Korean goes so quickly that I worry about getting left behind. |
| 9 | I feel more tense and nervous speaking in Korean than in my native language. |
| 10 | I am afraid that other people will laugh at me when I speak Korean. |
| ***L2 Willingness to Communicate (WTC)*** | |
| 1 | When you are given a chance to talk freely in Korean. |
| 2 | When you have a chance to speak in Korean in front of others. |
| 3 | When you have a group discussion in Korean. |
| 4 | When you have a chance to make a presentation in Korean in front of a large group. |
| 5 | When you find a friend standing before you in a line and have a chance to speak in Korean. |
| 6 | When you find an acquaintance standing before you in a line and have a chance to speak in Korean. |
| 7 | When you have a discussion in Korean with a small group of friends. |
| 8 | When you have a chance to talk in Korean within a small group of strangers. |

**Appendix B. Interviewee profiles**

| **Participant (P)** | **Sex** | **Age** | **Year** | **TOPIK** | **AI-IDLK Learning Focus and Purpose** |
| --- | --- | --- | --- | --- | --- |
| **P1** | Female | 22 | Year 3 | Advanced | Enhanced overall communicative competence and discourse organization through real-life simulations and pronunciation feedback |
| **P2** | Male | 20 | Year 2 | Intermediate | Improved interactional fluency and expression through scenario-based dialogues and integrated reading–writing tasks |
| **P3** | Female | 18 | Year 1 | Beginner | Developed basic linguistic accuracy through pronunciation correction, grammar practice, and core vocabulary use |
| **P4** | Female | 23 | Postgraduate | Intermediate | Strengthened functional language use in real contexts through error correction and contextualized expression practice |
| **P5** | Male | 19 | Year 1 | Beginner | Built foundational language competence through vocabulary learning and multimodal input activities (listening and reading) |
| **P6** | Female | 21 | Year2 | Advanced | Refined discourse development and language integration through diverse production and text-generation tasks |
| **P7** | Female | 20 | Year 2 | Intermediate | Enhanced communicative flexibility and coherence through grammar correction and scenario-based interaction |
| **P8** | Female | 23 | Year 4 | Advanced | Advanced logical organization and linguistic sophistication through academic writing and presentation revision tasks |
| **P9** | Male | 27 | Postgraduate | Advanced | Strengthened higher-order discourse performance and critical reasoning through topic-based discussions and data interpretation |

**Appendix C. Semi-Structured Interview Guide**

| 1 | Could you briefly introduce yourself? |
| --- | --- |
| 2 | What motivated you to start using AI in your Korean learning? |
| 3 | Have you ever taken an AI-based Korean language course? |
| 4 | In what ways or patterns did you mainly use AI? (e.g., speaking practice, grammar correction, free conversation, etc.) |
| 5 | What kinds of prompts did you mainly use when learning with AI, and why? |
| 6 | How did your experience with AI-IDLK differ from traditional classroom learning or textbook-based learning? |
| 7 | Before starting AI-based learning, what was the state of your speaking confidence and anxiety? |
| 8 | In what ways did the AI-IDLK environment influence your confidence? Please provide specific examples. |
| 9 | Did the confidence you gained through AI practice carry over into actual classroom interactions or conversations with teachers and peers? If so, why do you think that happened? |
| 10 | How did your speaking anxiety change while interacting with AI? Please explain the reasons as well. |
| 11 | Did you experience anxiety even while learning with AI? If so, in what specific situations did it occur? |
| 12 | When confidence and anxiety coexisted, which factor do you think played a greater role in determining whether you actually spoke? |
| 13 | How did your AI-IDLK experience influence your willingness to initiate real-life conversations in Korean? |
| 14 | After your AI-IDLK experience, did you notice any changes in your speaking behavior during classroom interactions or with peers? If so, how did it change? |
| 15 | Without your AI-IDLK experience, how do you think your current confidence, anxiety, and willingness to communicate would have been different? |
| 16 | Do you want to continue learning with AI in a self-directed way in the future? If so, why? |

**Appendix D. Interview coding examples**

| Themes | Codes | Examples of data extracts |
| --- | --- | --- |
| **L2 confidence enhancement pathway** | Psychological safety | I: When practicing Korean through AI-IDLK, was there anything that made you feel more comfortable speaking?  P3: Yes. AI doesn’t laugh even if I make mistakes, so I don’t have to worry about being wrong. Because of that, I felt less pressure and could keep trying to speak. |
|  | Repetitive production practice | 2) I: In what way did practicing Korean through AI-IDLK affect your Korean speaking?   P7: In class, speaking opportunities are limited, so it is hard to practice enough. In class, there are few opportunities to speak, but with AI, I can keep talking as much as I want, so my expressive ability has improved. Repeating this process made me feel more confident about speaking. |
|  | Personalized adaptive learning | 3) I: Did AI-IDLK help you in ways that were different from textbooks or ordinary classroom learning?   P4: Yes, very much. In textbooks there is only one correct answer, but AI gives accurate responses suited to my utterances… It is my one-on-one teacher. Because the responses matched my level and what I wanted to say, I felt more prepared to speak. |
|  | Sustainable accessibility | 4) P3: One important advantage was that I could practice whenever I needed to. I can practice Korean regardless of day or night. That made it easier to continue speaking practice consistently without being restricted by time or place. |
|  | Discourse agency & engagement | 5) **I:** What made you continue speaking through AI-IDLK for a longer time?   **P7:** Sometimes I became so immersed in the interaction that I forgot how much time had passed. **I enjoyed talking with AI so much that I kept speaking in Korean without being aware of time.** In those moments, speaking Korean felt natural rather than forced. |
| **L2 anxiety persistence pathway** | Uncertainty of AI responses | 6) **I:** Did practicing Korean through AI-IDLK ever make you feel anxious?   **P4:** Yes, especially when I later realized the AI response was not reliable. **AI said it was correct, but it turned out to be wrong. When my teacher later said something different, I felt that everything I had practiced was wrong and stopped speaking.** After that, I became more cautious and anxious during practice. |
|  | Uncontrollability of interaction | 7) **P5:** Sometimes the conversation did not go the way I expected. **When AI suddenly responds in a different direction while I’m studying, I lose the desire to continue speaking.** At those times, instead of feeling supported, I felt interrupted and frustrated. |
|  | Uncritical praise | 8) **I:** How did you feel about the feedback provided through AI-IDLK?   **P3:** At first, positive feedback felt encouraging, but later I began to doubt it. **AI keeps saying I did well no matter what I do, so I couldn’t tell whether it really meant I passed or it just overlooked my mistakes.** That uncertainty actually made me more anxious. |
|  | Gap with real interaction | 9) **I:** Even after practicing through AI-IDLK, did you still feel nervous or anxious when communicating with real people?   **P7:** Yes. **AI only answers what I ask, but when I talk with people, unexpected questions make me nervous.** So even though AI practice helped, I still felt anxious in actual interaction with people. |
